# SABR-BRIDGE: *S*tereotactic *AB*lative *R*adiotherapy *B*efore *R*esection to Avo*I*d *D*elay for Early-Stage Lun*G* Cancer or Oligom*E*ts During the COVID-19 Pandemic

**DOI:** 10.3389/fonc.2020.580189

**Published:** 2020-09-25

**Authors:** Biniam Kidane, Jonathan Spicer, Julian O. Kim, Pierre-Olivier Fiset, Bassam Abdulkarim, Richard Malthaner, David Palma

**Affiliations:** ^1^Section of Thoracic Surgery, Department of Surgery, Rady Faculty of Health Sciences, University of Manitoba, Winnipeg, MB, Canada; ^2^Department of Community Health Sciences, University of Manitoba, Winnipeg, MB, Canada; ^3^Research Institute in Oncology and Hematology, Cancer Care Manitoba, University of Manitoba, Winnipeg, MB, Canada; ^4^Division of Thoracic Surgery, Department of Surgery, McGill University, Montreal, QC, Canada; ^5^Research Institute of the McGill University Health Center, Montreal, QC, Canada; ^6^Department of Radiation Oncology, Rady Faculty of Health Sciences, University of Manitoba, Winnipeg, MB, Canada; ^7^Department of Pathology, McGill University, Montreal, QC, Canada; ^8^Division of Radiation Oncology, Department of Oncology, McGill University and Cedars Cancer Center, Montreal, QC, Canada; ^9^Division of Thoracic Surgery, Department of Surgery, Western University, London, ON, Canada; ^10^Lawson Health Research Institute, London, ON, Canada; ^11^Division of Radiation Oncology, Western University, London, ON, Canada

**Keywords:** lung cancer, nonsmall cell lung cancer, pulmonary metastases, SABR lung, SBRT (stereotactic body radiation therapy), COVID-19, lobectomy (lung), sublobar pulmonary resection

## Abstract

Surgical resection is the standard-of-care approach for early-stage non-small cell lung cancer (NSCLC). Surgery is also considered an acceptable standard infit patients with oligometastatic lesions in the lungs. The COVID-19 pandemic has led to worldwide issues with access to operating room time, with patients and physicians facing uncertainty as to when surgical resection will be available, with likely delays of months. Further compounding this are concerns about increased risks of respiratory complications with lung cancer surgery during active phases of the pandemic. In this setting, many thoracic oncology teams are embracing a paradigm where stereotactic ablative radiotherapy (SABR) is used as a bridge, to provide radical-intent treatment based on a combination of immediate SABR followed by planned surgery in 3–6 months. This pragmatic approach to treatment has been named SABR-BRIDGE (Stereotactic ABlative Radiotherapy Before Resection to avoId Delay for early-stage lunG cancer or oligomEts). This term has also been applied to the pragmatic study of the outcomes of this approach. In this paper, we discuss the standards of care in treatment of early-stage (NSCLC) and pulmonary oligometastases, the impetus for the SABR-BRIDGE approach, and the controversies surrounding assessment of pathological response to neo-adjuvant radiation therapy.

## Introduction

Surgical resection is the standard-of-care approach for early-stage non-small cell lung cancer (NSCLC) and for many fit patients with oligometastatic lesions in the lungs. The COVID-19 pandemic has led to worldwide issues with access to operating room time, with patients and physicians facing uncertainty as to when surgical resection will be available, with likely delays of months. In this setting, many thoracic oncology teams are embracing a paradigm where stereotactic ablative radiotherapy (SABR) is used as a bridge, to provide radical-intent treatment based on a combination of immediate SABR followed by planned surgery in 3–6 months. This pragmatic approach to treatment has been named SABR-BRIDGE (***S***tereotactic ***AB***lative ***R***adiotherapy ***B***efore ***R***esection to avo***I***d ***D***elay for early-stage lun***G*** cancer or oligom***E***ts). This term has also been applied to the pragmatic study of the outcomes of this approach. In this paper, we discuss the standards of care in treatment of early-stage (NSCLC) and pulmonary oligometastases, the impetus for the SABR-BRIDGE paradigm, and the controversies surrounding assessment of pathological response to neo-adjuvant radiation therapy.

## Standard of Care Treatment: Surgery

For decades, surgery has been the cornerstone of cure for patients with NSCLC. Through numerous technical innovations and iterative improvements in care pathways, the vast majority of early stage lung cancers are operable by minimally invasive techniques resulting in a short hospitalization and a risk profile that has continued to improve over the years (1, 2). Most recent data indicate that pulmonary resection is associated with a 1.3% 30-day mortality risk and a major morbidity risk of 7.9% based on large-scale report from the Society of Thoracic Surgeons' General Thoracic Surgery Database (3). While data are still pending on the long term oncologic impact of lobar vs. sublobar resections, we now know that the short-term perioperative outcomes are almost identical for patients with stage I lung cancer, with the exception of higher rates of prolonged air leak after segmentectomy (2, 4). Nonetheless, the advantages of surgery for early stage lung cancer are numerous: definitive diagnosis without risk of sampling error, low local recurrence rates, excellent regional control, and definitive nodal staging with opportunities for adjuvant systemic therapy in the context of node-positive disease.

Surgery for early stage lung cancer can be viewed as a higher risk but higherreward proposition for patients as compared to less invasive techniques like SABR. In general, the higher risks with surgery are in the peri-operative short term. Furthermore, not all patients have sufficient pulmonary reserve and are physiologically fit to undergo curative lung surgery (5). Several factors have traditionally been known to impact risk such as age, pulmonary function, co-morbidities, surgeon/hospital volume, and minimally invasive surgical approaches (6). Unfortunately, the impact of the SARS-CoV2 pandemic on the risk of pulmonary surgery is not known. While it is clear that cancer patients have higher risk of death from COVID-19 compared to the general population, how this relates to the impact of curative lung surgery has not been well-documented (7). Limited data are available, but it is clear from recent studies that any type of surgery performed on a patient infected with COVID-19 may be associated with an unacceptably high mortality risk in the context of elective surgery (8, 9). Although some data indicate that surgery is not associated with a higher risk of mortality, these are still early days in our understanding of COVID-19 and there are widely conflicting retrospective data available at this time (10, 11). Due to the risk profile presented by surgery and the growing body of data indicating effective long-term cancer control for inoperable SABR patients, the question of whether SABR is an equivalent or better modality of cure to surgery, even in operable patients, has been raised for many years (12). Thus far, the thoracic oncology community has failed to answer this important question. Fortunately, there are a number of promising clinical trials underway comparing both modalities head to head (13–15). The results of these studies are many years away and unfortunately will not help us cope with the global health care crisis resulting from the COVID-19 pandemic.

With regards to pulmonary metastasis, surgical resection has also been the favored modality of pulmonary local control in patients who are physiologically fit and have sufficient pulmonary reserve (16). That being said, the most recent prospective clinical trial for lung metastases are with SABR (mostly in the form of basket trials). Though data are still lacking in terms of well-structured head to head randomized comparisons of systemic therapy only vs. systemic therapy with pulmonary metastatectomy for several primary histologies, the overwhelming historical data indicate that survival of patients who undergo surgical metastatectomy are undeniably better than those who do not (17). Whether this is due to the intrinsic biology of the disease for patients who happen to be well enough to have a pulmonary metastatectomy or if the act of metastatectomy is directly responsible for this noted improvement in survival remains an open question. However, well-designed clinical trials investigating the impact of local consolidation after systemic therapy in oligometastatic disease clearly indicate that there is a therapeutic effect to local consolidation for numerous primary cancer histologies (18, 19). Nonetheless, while non-surgical local consolidation therapies such as SABR are thought to drive the survival effect in these trials, the local pulmonary control estimates suggest that non-surgical approaches are more prone to recurrence/progression of disease which may not be as frequent when lesions are surgically removed (20). In one study, SABR local recurrence at 5 years was 37% whereas it was only 18% for wedge resection (20). On the other hand, some studies suggest more promising local control rates with SABR; for example, the phase 2 single-arm trial SABR-COMET showed that only 3% of treated pulmonary metastases had local recurrences (18). In truth, the fact remains that all data surrounding these clinical scenarios are still murky and highly prone to bias. Thus, the state of the evidence suggests that both surgery and SABR are acceptable standards for oligometastatic disease.

## Standard of Care Treatment: SABR

Stereotactic Ablative Radiotherapy for early-stage (T1-T2N0M0) NSCLC is a clinically proven and accepted standard of care for patients who are surgically inoperable as well as for patients who refuse surgical resection (21). Modern SABR techniques have been in clinical use for over a decade and typically integrate highly conformal dose distributions to the target tumor (achieved by intensity-modulated radiotherapy or volumetric modulated arc therapy), ablative doses of radiotherapy with biological equivalent doses of ≥100 Gy delivered in a small number of fractions, tumor motion mitigation strategies (such as 4-dimensional CT simulation scans, respiratory gating, or breath holds), and patient setup techniques which minimize inter/intra fraction setup errors (including cone beam CT scan based image guidance, fiducial markers, or real time electromagnetic transponder beacon guidance) (22, 23).

A number of prospective, multicenter clinical trials have demonstrated excellent local control of early-stage NSCLC with minimal levels of acute and late toxicity using a variety of fractionation options including 54–60 Gy in three fractions, 48 Gy in four fractions, and 30–34 Gy in one fractions for tumors which are located peripherally (outside of 2 cm from the proximal bronchial tree) (24–27) SABR is also feasible to deliver to tumors which are located within 2 cm of the proximal bronchial tree (central or ultracentral tumors), through the use of more cautious dose fractionation options including 60 Gy in eight fractions and 50 Gy in five fractions (28, 29).

Although there is randomized evidence to support the superiority of SABR to conventionally-fractionated lung radiotherapy, attempts undertaken to date comparing SABR to surgical resection in the randomized sphere have suffered slow accrual leading to early trial closure (30). Although pooled analyses have been performed comparing SABR to surgical resection, these analyses were underpowered to draw any definitive conclusions (12). As described in the previous section above, further research to address this clinical equipoise is currently underway (13–15).

As described in the previous section, SABR is also employed for the management of pulmonary oligometastatic disease with emerging evidence suggestive of improved survival outcomes amongst those treated with SABR vs. the standard of care (18).

## Challenges during COVID19 Pandemic

The COVID-19 pandemic has required all of us to make unprecedented systematic and *ad hoc* triage decisions about the delivery of care to cancer patients. Surgical societies including those that are involved in the treatment of lung cancer underwent development of rapid consensus statements based mostly on existing evidence and consensus expert opinion, with the ultimate aim of providing guidance for systematic triaging during different anticipated phases of the pandemic (31). The development of such triage guidelines for lung surgery was led by and included the following groups: American College of Surgeons Commission on Cancer, American Association for Thoracic Surgery, Society of Thoracic Surgeons, Thoracic Surgery Research Outcomes Network (ThORN) (31). The consensus discussions and statement were informed by evolving knowledge and circumstances. Thus, the guiding principles were to provide an objective and transparent framework to triage patients according to the state of the pandemic and healthcare resources. In all phases of the pandemic, the recommendation was to either offer alternative options (i.e., SABR) or to delay/defer treatment of patients with early stage lung cancer or oligometastatic disease to the lungs (31).

Early in the COVID-19 pandemic, a working group of 32 radiation oncologists representing the American and European Radiation Oncology societies (ASTRO and ESTRO, respectively) completed a rapid modified Delphi consensus project in order to provide guidance to radiation oncologists (32). The recommendations were stratified into two possible pandemic phases: an early pandemic phase, also called “contingency standard of care,” where radiation resources are available but risks to the patient due to COVID-19 must be minimized (32). These risks include the possibility of acquiring a COVID-19 infection during a hospital visit, and the dangers related to immunosuppression from anti-cancer treatment (32). The second, later pandemic phase, as called “crisis standard of care,” refers to a situation where treatment capacity is lost (e.g., due to staff illnesses, facility closures, and/or inability to maintain equipment) and patients must be triaged since not all can be treated (32). The ESTRO-ASTRO consensus group was unanimous in recommending SABR in a patient with stage I NSCLC referred by a surgeon because of operating room closures or delays (32). Their group also supported delivering SABR as a single fraction if the treating physician wished to reduce the number of visits in the early pandemic phase (25, 26, 32).

Two recent randomized trials have indicated promising results when using a single-fraction SABR approach (one dose of 30–34 Gy), with similar toxicity and oncologic outcomes compared to a 3-fraction (60 Gy) regimen and a 4-fraction (48 Gy regimen) (25, 26). During the COVID-19 pandemic, the single-fraction SABR approach has the advantage of reducing visits to radiotherapy departments down to only two: one visit for CT simulation, and one visit for treatment (25, 26, 32).

## SABR as a Bridge to Surgery

The use of SABR as neoadjuvant therapy prior to surgery was evaluated in the MISSILE phase II trial (33). In this trial, 40 operable patients with T1-2N0 NSCLC were enrolled (33). Patients were treated with SABR followed by surgical resection 10 weeks later (33). The primary endpoint was the pathologic complete response (pCR) rate, with secondary endpoints including toxicity and efficacy (33). The trial demonstrated reasonable toxicity outcomes, similar to studies reporting on surgery alone, no 30- or 90-day mortality, and a pathologic complete response rate of 60% (33). Neoadjuvant SABR provides potential advantages as a therapy to bridge until definitive surgery, including potentially sterilizing the tumor.

The current COVID-19 pandemic has resulted in a re-evaluation of all surgeries and the safety of pulmonary surgery has been a major focus given the natural history of SARS-CoV2 infection. In many centers, elective surgeries are canceled for at least a window of 3–4 months, and cancer surgeries are being triaged to identify cancers that can be safely deferred for 3–4 months without a major risk of upstaging or adverse outcomes. As the phases of the pandemic response escalate, patients with early stage lung cancers and lung oligometastases would fall into this category. Even when elective cancer surgeries are resumed at varying rates worldwide, it may not mean that these patients will immediately start getting surgery. Data-driven modeling suggests that in excess of 28 million surgeries were delayed or canceled globally during the first 3 months of the COVID-19 pandemic. This data, which used Bayesian regression of responses from 190 countries, also suggests that the number of cancer surgeries delayed or canceled were in excess of 2.3 million (34). Thus, this is the backlog of surgeries that we all must struggle to rectify, while still encountering the reality of the usual ongoing need to treat new incident cases. In many health systems globally, it was a challenge to deliver timely care to patients with new incident cancers even before the pandemic. This is the context upon which the harrowing backlog of cancer surgeries has now been superimposed.

Thus, the favored approach in these patients by some multidisciplinary teams is to deliver SABR as the initial modality of treatment, with re-evaluation for surgical resection thereafter, rather than providing no treatment for 3–4 months or proceeding with an aerosol-generating lung surgery (thereby putting patients, health care workers and the health system at risk of deleterious outcomes during this pandemic). Because surgery is still the standard of care for operable patients, and given the findings of the MISSILE trial, these patients would then be offered surgical resection (lobectomy or sublobar resection with nodal dissection) in 3–6 months or once the acute phases of this pandemic subside. Even as elective surgeries are being resumed, many considerations inform which cancers should be prioritized for surgery. One potential consideration is whether that cancer has any viable proven alternatives. Whereas many surgically-treated cancers do not have proven “equivalent” alternatives, early stage lung cancer and lung oligometastases fortunately have an alternative that has been proven to be effective in the short-to-medium term (12).

Whether surgical resection after SABR is absolutely required is unknown. The definition of local control for SABR trials remains challenging given the paucity of pathological data. Many patients achieve long-term survival after SABR alone, but since SABR alone has not been proven to be non-inferior to the gold-standard approach (i.e., surgical resection), omitting surgery or delaying beyond 6 months may be risky. There are several reasons why a planned surgical resection may be preferred to a “watch-and-wait” approach after SABR. First, there is substantial difficulty in assessing radiologic outcomes after SABR, because asymptomatic radiation-induced fibrosis, which occurs in nearly all patients, can be difficult to distinguish from recurrence (35). Second, 40% of patients in the MISSILE trial had viable cells detected on pathology after SABR, and although the reproductive potential of those cells is unknown, caution is warranted (33). Thirdly, delaying resection until definite evidence of progression is available (based on serial imaging, including CT, PET/CT, and also biopsy) may put a patient at higher risk of metastasis, and may lead to more difficulty in resection due to fibrosis. Finally, surgical resection allows for assessment and removal of the regional lymph nodes, which are expected to contain occult microscopic metastases in 10–20% of patients (36).

This also provides secondary research benefits, with an ability to assess outcomes with a SABR-BRIDGE approach, including oncologic outcomes, toxicity, and provide further data on the pathologic complete response (pCR) rate after SABR. Given that the COVID-19 pandemic may be of several months to years in duration, if future peaks occur that require operating room closures, these results will be useful to guide treatment.

## Controversy Regarding Pathological Response After SABR

Pathological response rate post-SABR is a source of controversy. Tumor cell viability in the MISSILE trial was assessed using standard hematoxylin-eosin staining and morphologic appearance of tumor cells on microscopy (33). The investigators reported a pCR rate of only 60% at 10 weeks post-SABR, which may not correlate with the high local control rate reported in several SABR published studies (33). These findings from MISSILE study highlight that response to SABR is poorly understood and underline the need to investigate further this response at the cellular and biological level.

The current radiobiological models of cell death post-SABR support that in addition to cell apoptosis, mitotic catastrophe is the primary cause of radiation-induced cell death in solid cancers (37). Radiation-induced DNA damaged cells initiate mitosis prematurely, leading to mitotic catastrophe (37). After SABR exposure, several cell divisions can occur before sufficient unrepaired DNA damage is accumulated to trigger mitotic cell death (37). This delayed cell death is likely the underlying mechanism for the delayed response often observed in solid tumors post-SABR (38). In this context, the choice of time point to assess cell death after SABR treatment is critical. Murine model studies have shown that tumor cells post-SABR can appear viable on histopathology but in fact are senescent from severe lethal DNA damage (39). Comparable evidence is reported in anal canal cancer treated with concomitant chemo-radiotherapy (CRT), wherein many patients with partial response (PR) at 11 weeks were demonstrated to develop complete response (CR) by 26 weeks (40). However, it is unclear if these findings can be directly translated to SABR, where the radiobiological effect induces both direct and indirect effects in controlling the tumor. While direct effects are the results of SABR-induced DNA damage in cancer cells leading to their death at various points after initial irradiation, the indirect effects include but not limited to tumor vasculature damage and priming of host anti-tumor immune response (41). Thus, it is possible that early assessment of pathologic response at 10 weeks post-SABR is poorly representative of the actual SABR damage and clonogenic cell survival response.

In a recent study using an orthotopic murine model of NSCLC, NSCLC was induced in 11 rats, of which five were assigned to observation and six received a SABR dose of 34 Gy in one fraction (39). Animals were sacrificed at different time points (10, 30, and 60 days post-SABR) to evaluate radiologic and histologic responses to SABR longitudinally (39). Radiologically, 4/6 animals had radiologic CR with disappearance of tumor on imaging within 30 days post-therapy, one had partial response, and one had radiologic progression (39). Interestingly, radiologic responses were found to match the observed pathologic responses: the four animals with CR had radiation-induced pneumonitis upon histology, and moderately differentiated mucinous adenocarcinoma was present in the two tumors that showed either partial response or progression (39). Although not directly related to the discussion regarding local pCR, it should be noted that 50% of animals with pCR developed metastatic disease, and cells surviving radiation were shown to have more invasive capacity and a more aggressive gene expression signature. This suggests clonal selection forces that may have deleterious effects at the systemic level.

After two decades of clinical practice, the randomized evidence base regarding SABR remains in its early stages, especially when compared to surgery for operable early-stage NSCLC patients. Furthermore, radiobiological models utilized in conventional fractionation schemes may not apply to SABR regimens (42). Data on neo-adjuvant SABR and assessment of pathological response post-SABR are sorely lacking, assessment of pCR is challenging, and the best timing for evaluation of pCR after SABR is unclear. The rat orthotopic model of human NSCLC adenocarcinoma can effectively mimic the heterogeneity of lung cancer biology and response to neo-adjuvant SABR observed in human tumors (39). It is therefore imperative to develop pre-clinical animal models of lung cancer in order to elucidate the molecular mechanisms of radio-resistance, tumor response post-SABR, and how to select those patients for immediate surgery or deferred surgery after SABR. The MISSILE study highlights the critical need for uniform criteria for pathological assessment of efficacy of SABR and the need to develop new biomarkers to assess residual tumor post-SABR both in surgical specimens and non-invasively without need for tissue sampling.

## Pathological Assessment After Neo-Adjuvant Therapy

Radiation therapy leads to formation of double strand breaks in DNA and loss of reproductive ability. The net response of tumors depends on classical 5 “R's” or radiobiology: repair of DNA, radiosensivity, reoxygenation, and redistribution of cells in the cell cycle (43). However, SABR may provide alternative mechanisms such as vascular damage and deterioration of the intratumor environment leading to tumor cell death (41). The resulting effects translate to varying histopathological features. The post-radiation microscopic landscape includes areas of giant irregular tumor cells, tumor necrosis and apoptosis, fibrosis, and inflammation. Inflammation can consist of varying degrees of acute and chronic inflammation and even collections of foreign body giant cells with cholesterol clefts (xanthogranulomatous response). Surrounding vessels show vascular damage and regenerative changes with sites of vasculitis and thrombus formation. The surrounding lung parenchyma shows varying degrees of radiation pneumonitis, which can confound radiologic prediction of patient prognosis (44). Findings include interstitial fibrosis, inflammation, organizing pneumonia, hemorrhage, and reactive atypia of the pneumocytes (45).

**Figure 1:**
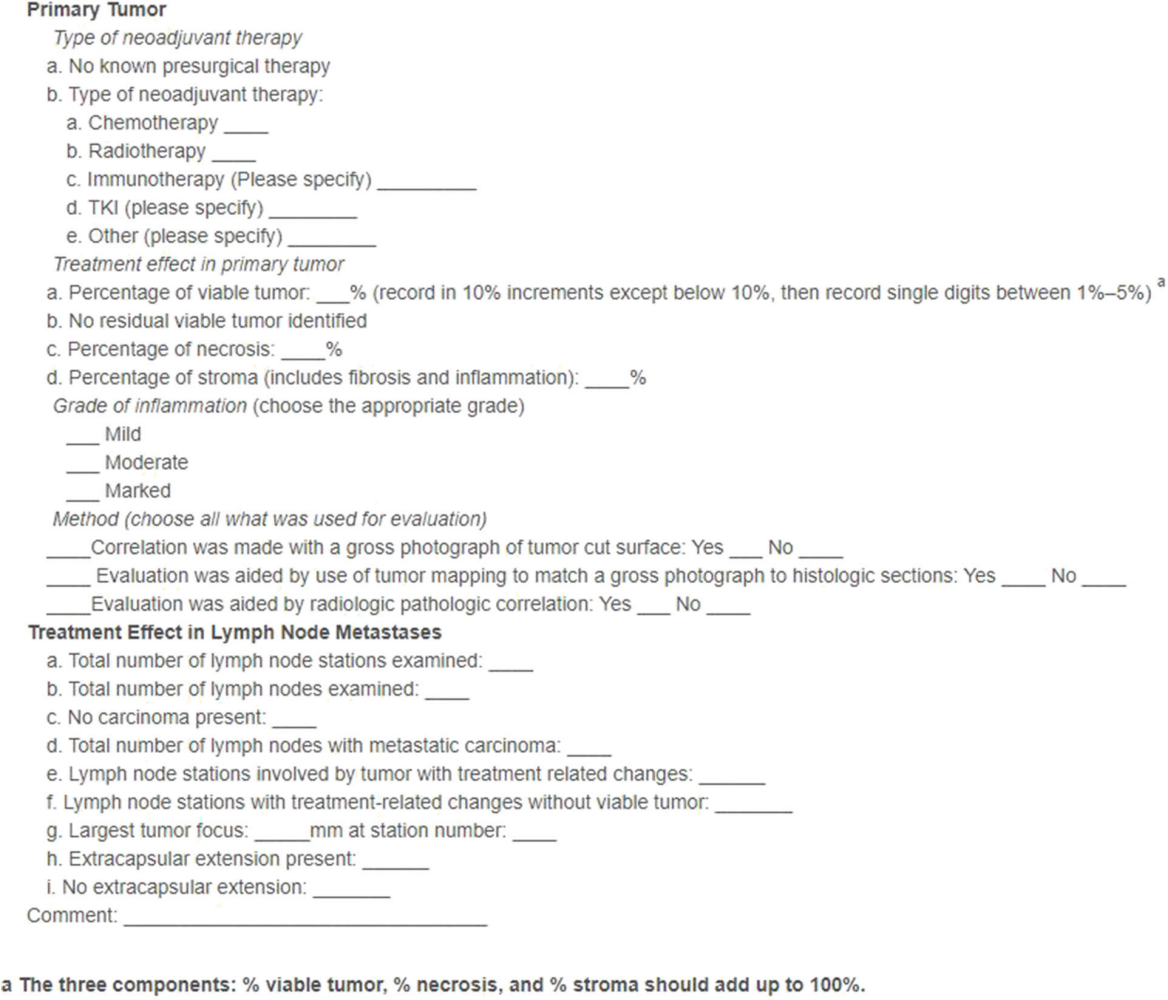
IASLC-recommended synoptic template for pathological reporting on resected lung cancers after neoadjuvant therapy. (Reprinted from J ThoracOncol. 2020;15(5); Travis WD, Dacic S, Wistuba I, Sholl L, Adusumilli P, Bubendorf L, et al. IASLC multidisciplinary recommendations for pathologic assessment of lung cancer resection specimens after neoadjuvant therapy*.J Thorac Oncol*. (2020) 15:709–40. doi: 10.1016/j.jtho.2020.01.005, with permission from Elsevier).

Viable areas of cells are also present after neo-adjuvant therapy and are among the most significant prognostic factors (46, 47). Precise endpoint definitions and standard methodologies are lacking, and thus the International Association for the Study of Lung Cancer (IASLC) has released recent expert-led guidelines and definitions to address these issues (48). These are aimed as guidance for clinical trials but may be incorporated into clinical practice. The definitions and proposed methodologies are therapy agnostic and thus can be applied to histopathological assessment after neo-adjuvant SABR. Complete pathologic response (cPR) is defined as no viable tumors after complete evaluation of the lung cancer specimen and sampled lymph nodes (48). A major pathologic response (mPR) is defined as less than or equal to 10% of viable tumor vs. size of the tumor bed (46). The IASLC guidelines provide a recommended synoptic template for pathological reporting on resected lung cancers after neoadjuvant therapy (48) (Figure 1). Our centers have adopted this recommended synoptic reporting and all centers using the SABR-BRIDGE approach are encouraged to do so.

Recommendations for gross assessment and sampling first includes identification of the tumor bed, measuring it in three dimensions, and sectioning along the maximum dimension of the specimen and its relationship to the margins (48). Photographs are key for documentation of submitted sections, distance to margins, estimation of gross necrosis, and correlation with microscopic findings. The tumor bed should be submitted in toto if ≤ 3.0 cm and if larger an entire cross-section of the tumor should be evaluated (48). Sections should include a rim of 1.0 cm of tissue beyond the tumor bed for evaluation of the surrounding lung parenchyma (48). Histological assessment of the effect includes identifying the true tumor beds and measurement of the percent viable tumor, stromal tissue (both the intratumoral inflammation and fibrosis), and amount of necrosis (48). The final pathological response is then correlated to the gross findings.

Several issues are present in evaluation of the response of neoadjuvant therapy. The optimal time to resection and evaluation of cPR/mPR from last radiation dose is not yet determined and must be balanced with the time point of maximal surgical benefit (49, 50). There may also be issues with reproducibility as the assessment may be affected by inter-observer subjectivity. The 10% threshold for mPR has been put into question for neoadjuvant chemotherapy as it may apply to squamous carcinoma but an mPR of 65% may better predict response for adenocarcinomas (51). Whether varying thresholds for mPR applies for neoadjuvant radiotherapy is to be determined. Moreover, the prognostic significance is unclear for a tumor with tumor bed mPR but with viable metastatic carcinoma cells found in the regional lymph nodes. Similarly, pathological stage assignment categories in neoadjuvant surgical resections need to be determined.

The SABR-BRIDGE approach facilitates the ability to address many unanswered important questions. Sampling of the tissues at the onset of the trial with comparison with post-treatment surgical resections can determine key determinants of resistance and promotion of anti-tumor inflammatory environments. Within the capacity of treatment centers, optimal banking protocols will be put in place with the ability to create a neoadjuvant biorepository, cell culture, patient-derived organoids, and patient-derived xenografts. Data collection initiatives will also be in place to allow for radiologic-pathologic correlation.

## Pragmatic Adoption of SABR-Bridge Approach

The following sections outline the basic principles of the pragmatic SABR-BRIDGE approach and data that will be collected as part of this approach.

### Eligible Patients

1. Histologically confirmed lung cancer, lung oligometastases, or clinically-determined lung malignancy (PET-avid or growing lesion in a high-risk patient) that would have otherwise been treated with resection.
2. Tumor size ≤ 5 cm.
3. No evidence of nodal disease (N0) or distant metastases based on imaging (i.e., N0 and M0).
4. ECOG performance status 0–2.
5. Life expectancy >6 months.
6. Adequate pulmonary for resection as determined by the treating surgeon.
7. No contraindications to radiation or surgery, in the opinions of the treating physicians.

### Staging Investigations

1. Standard-of-care staging investigations are ideal (i.e., PET/CT, and if T2 or central disease then brain imaging).
  - However, standard staging investigations may not be fully available and clinicians will weigh the pros and cons of waiting until staging is available vs. proceeding with treatment.

2. Staging of the mediastinum as per the standard of care.
  - Due to the risks of aerosol generation with bronchoscopy and the reliance on use of high-level PPE for these, clinical mediastinal staging via PET-CT will be accepted.
  - Invasive mediastinal staging (Mediastinoscopy or EBUS/EUS) will be used in patients with abnormal mediastinal or hilar nodes by CT or PET criteria.

3. Pulmonary function tests as per local institutional standard of care.

### SABR Fractionation

SABR is to be delivered using standard institutional guidelines, using standard immobilization and motion management. Due to the potential for variation of availability of radiotherapy resources during the COVID19 pandemic, and to minimize the number of trips that patients take to the cancer center for SBRT treatment, radiation oncologists should attempt to deliver radiotherapy in as few fractions as feasible. With this in mind, we ask that radiation oncologists strongly consider the use of single fraction SBRT over multifraction schedules to the greatest extent possible for non-central tumors. Central tumors (within 2 cm of proximal bronchial tree or mediastinum, or near brachial plexus) may be treated with 50 Gy in five fractions or 60 Gy in eight fractions.

Standard dose constraints from clinical trial protocols will be used (Appendix).

Acceptable fractionations for non-central tumors:

1. 30–34 Gy in 1
2. 45–55 Gy in 3–5 fractions (e.g., 54/3, 48/4, and 55/5)
3. 60 Gy in eight fractions.

### SABR Volume Definitions and Prescription

The gross tumor volume (GTV) will be defined as the visible tumor on CT imaging ± PET, and an internal GTV (iGTV, also known as the internal target volume) will be defined as the GTV from all phases of respiration, if gating is not used. No additional margin will be added for microscopic spread of disease. A Planning Target Volume (PTV) margin of 5 mm will be added. Organs at risk within 5 cm of the PTV will be contoured.

Doses are prescribed to ~80% isodose line surrounding the PTV, resulting in a hotspot of 120–140%; the latter should fall within the iGTV. Ninety-five percent of the PTV should be covered by the prescription dose, and 99% of the PTV should be covered by 90% of the prescription dose. Several non-overlapping 6/10 MV beams (on the order of 7–11 beams) or 1–2 VMAT arcs combined possibly with a few non-coplanar beams should be utilized. Non-coplanar beams can be used to reduce 50% isodose volume for un-gated treatments.

### Surgery

Surgery will aim to occur at 12–24 weeks following SABR, but may be done earlier or later depending on the status of the COVID-19 epidemic. There should be at least one diagnostic CT chest done prior to resection. Patients can elect to decline surgery, with their surveillance (clinical and radiographic) recorded. Reason(s) for declining surgery will be documented. Surgery will consist of a lobectomy, or sublobar resection, and may employ either an open approach or a minimally-invasive approach. Surgical sampling of the at-risk hilar and mediastinal nodes will be done at the time of resection; sampling of three N2 and one N1 nodal station will be recommended and ideal.

### Pathology Assessment

Pathology assessment will be accomplished according to standard institutional guidelines, however, centers are encouraged to use the IASLC recommended synoptic template for pathological reporting on resected lung cancers after neoadjuvant therapy (Figure 1).

### Adjuvant Treatment

Adjuvant chemotherapy will be delivered as per routine standard practice. Any patients with pathologic node-positive disease (N1, N2, or N3) will be referred for an opinion from a medical oncologist. For patients with N2 or N3 disease, adjuvant radiotherapy to the mediastinum may be considered as per institutional practice, provided there is minimal overlap with the SABR dose distribution.

### Follow-Up

Patients who undergo resection will be followed as per standard of care practice, which may include CT imaging of the chest every 6 months. In a setting where a patient does not undergo resection, the patient would be followed by the radiation oncologist.

### Research Data Collection

**Figure 2:**
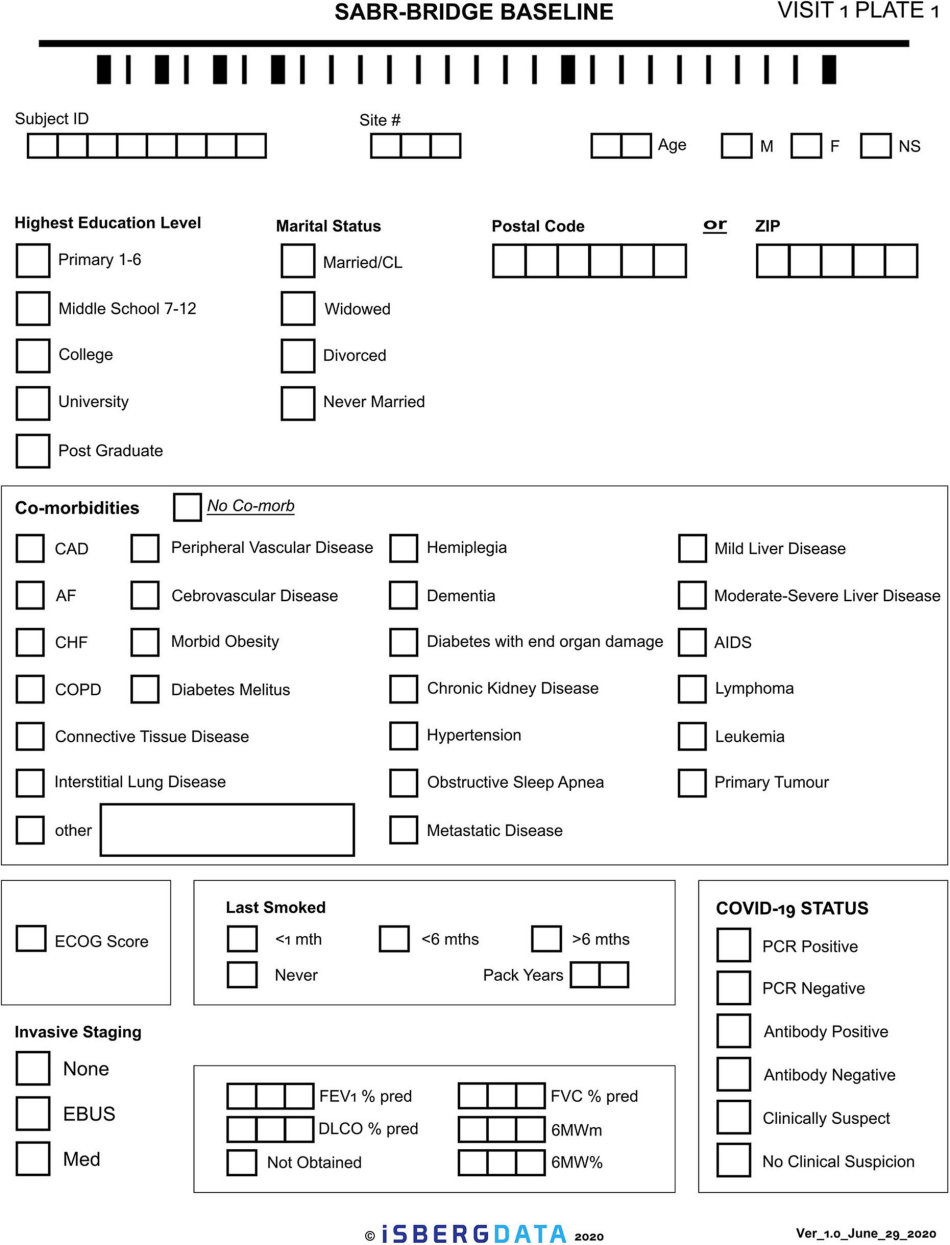
Baseline data collection form.

**Figure 3:**
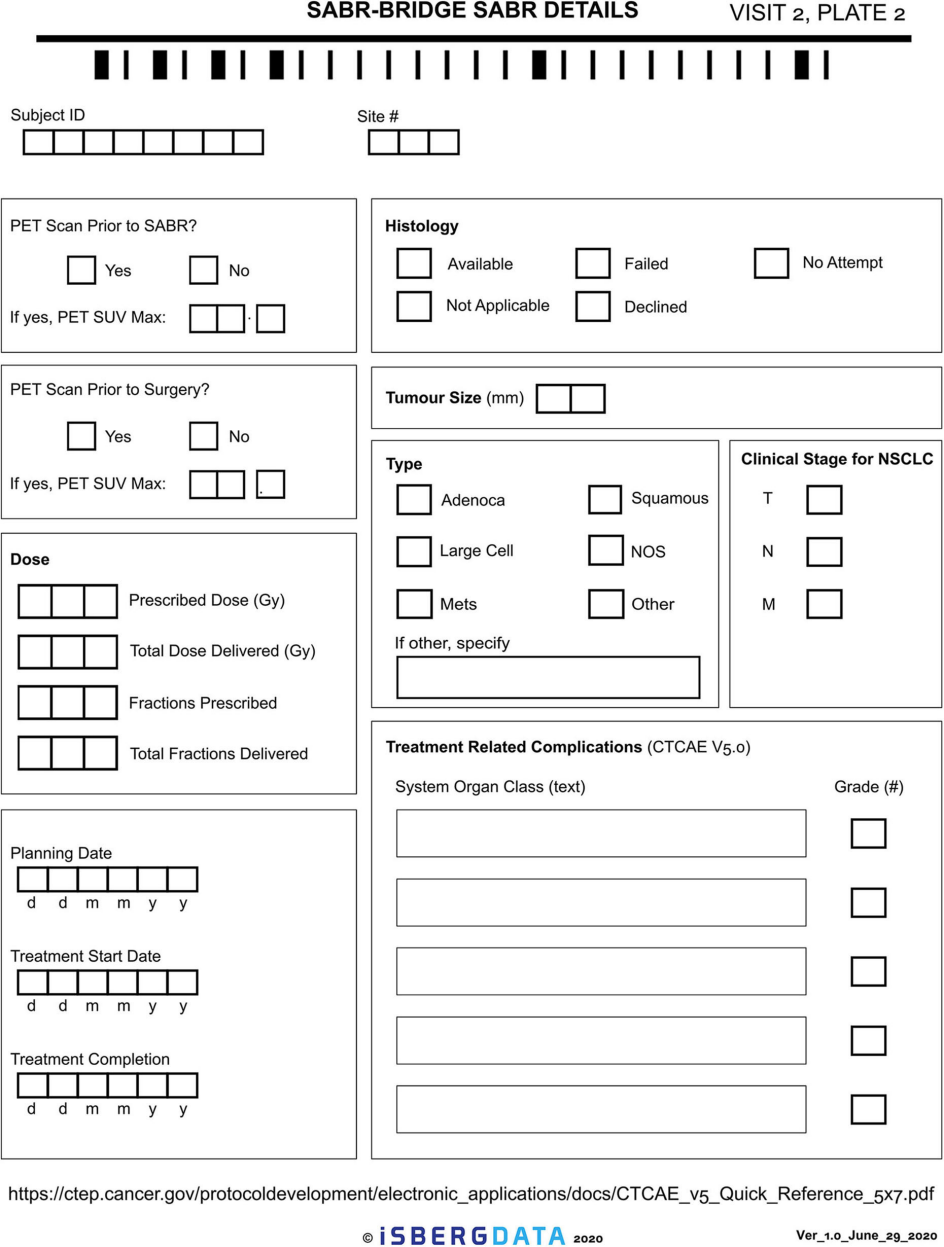
SABR treatment data collection form.

**Figure 4:**
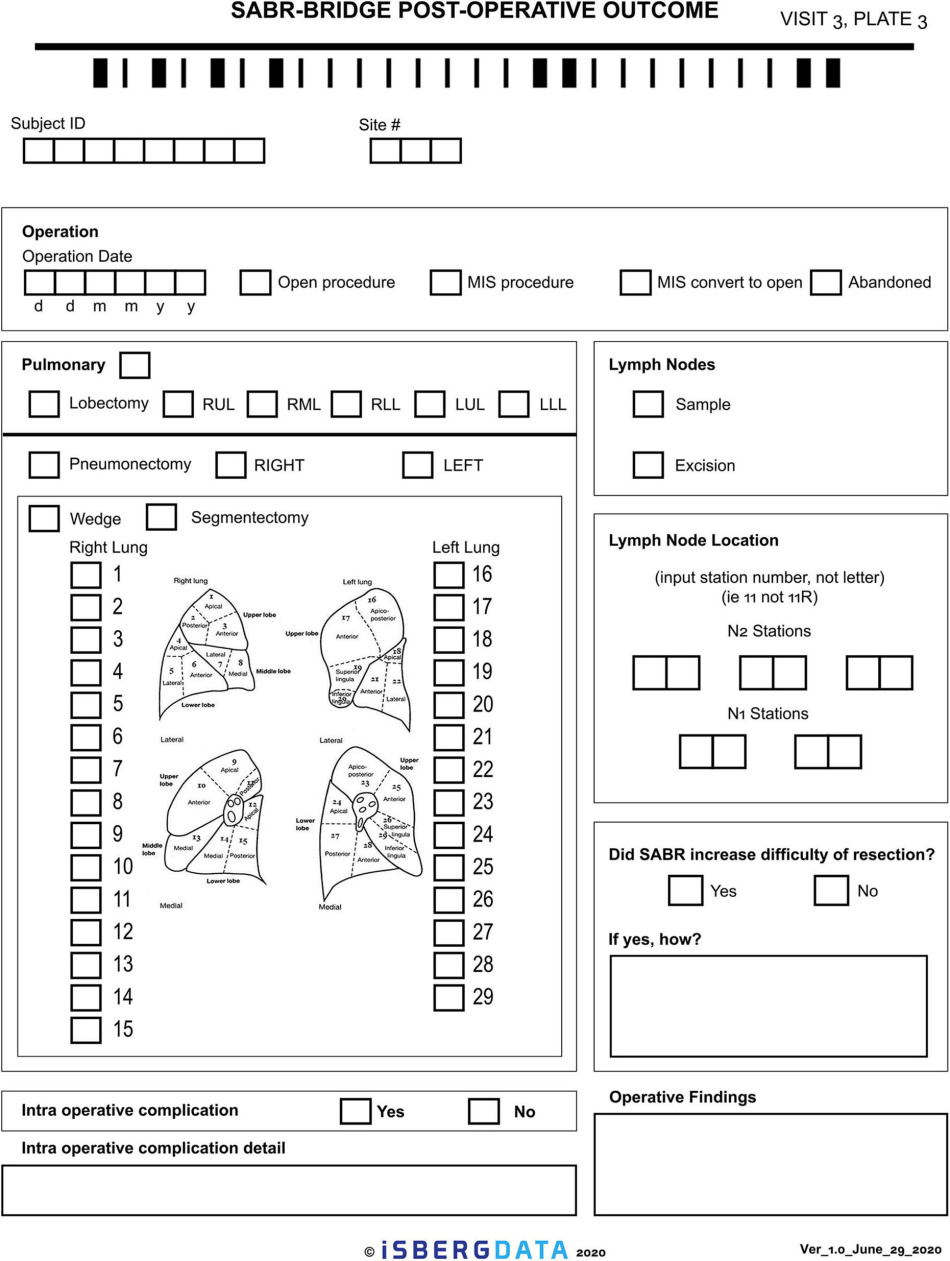
Operative data collection form.

**Figure 5:**
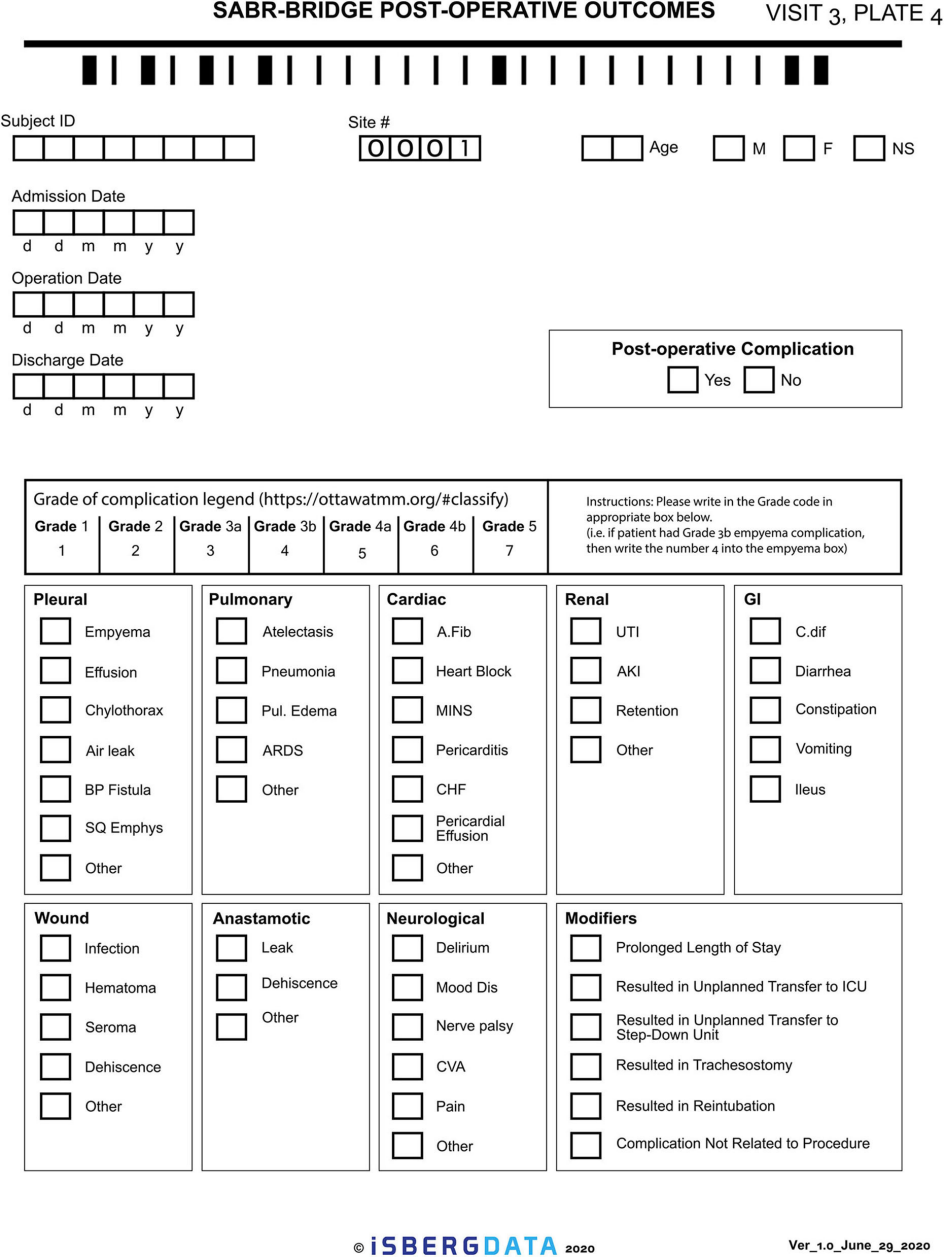
Post-operative outcomes data collection form.

Given the novelty of this treatment approach, it is important to study and report on the outcomes. The research aspect of this will aim to collect real-world data, either retrospectively or prospectively, on patients who are treated with the SABR-BRIDGE approach, including any who may not elect to ultimately undergo resection. Centers will hopefully elect to pool their data in the future. Data elements to be collected are shown in Appendix 1. Figure 2 shows the Baseline data collection form. Figure 3 shows the SABR treatment data collection form. Figure 4 shows the operative data collection form. Figure 5 shows the post-operative outcomes data collection form.

The names and personal information of study participants will be held in strict confidence and de-identified in the event of multi-center data pooling.

## Conclusion

The world as we know it has been fundamentally changed in last few months and it is not likely to return to normal in the short or intermediate term. Our current situation is characterized by a combination of concern around the potentially increased short term risk of lung surgery in the midst of this pandemic with the fear that patients may be offered sub-optimal cancer control when SABR is offered as the only modality. Indeed, under normal circumstances the patients potentially eligible to participate in our study would have been offered surgery as the favored modality of cure or local consolidation. Although the pandemic has broad devastating and fatal consequences, the opportunities presented by recent events have forced us to reassess alternative options that offer both curative treatment while also increasing our basic scientific understanding of lung cancer and pulmonary oligometastatic treatments. Perhaps most importantly, the pandemic has forced us to consider alternative therapeutic strategies that leverage the known clinical advantages of all available therapeutic platforms—in this case, offering SABR as a bridge to surgery in early stage lung cancer and pulmonary oligometastasis.

## Author Contributions

Conception or design of the work: BK, JS, and DP. Data collection: BK, JS, JK, P-OF, BA, RM, and DP. Data analysis and interpretation: BK, JS, JK, P-OF, BA, RM, and DP. Drafting the article: BK, JS, JK, P-OF, BA, RM, and DP. Critical revision of the article: BK, JS, JK, P-OF, BA, RM, and DP. Final approval of the version to be published: BK, JS, JK, P-OF, BA, RM, and DP. All authors contributed to the article and approved the submitted version.

## Conflict of Interest

The authors declare that the research was conducted in the absence of any commercial or financial relationships that could be construed as a potential conflict of interest.
