# Supplementary material for: SABR-BRIDGE: Stereotactic ABlative Radiotherapy Before Resection to AvoId Delay for Early-Stage LunG Cancer or OligomEts During the COVID-19 Pandemic
Source: Front Oncol. 2020 Sep 25;10:580189. doi: 10.3389/fonc.2020.580189 (PMC7544973; doi:10.3389/fonc.2020.580189)
Supplement: Supplementary file 1 [file Table_1.doc]

# Appendix 1 : Data Collection Elements

**Baseline/Pre-treatment**

1. Age at time of diagnosis
2. Sex
3. Tumour laterality and lobe
4. AJCC 8 ed stage (TNM)
5. Biopsy confirmed (yes/no)
   1. if confirmed, pathological subtype (Adenoca, SCC, Large Cell, NOS)
6. FEV1, DLCO
7. ECOG Performance Status
8. PET scan prior to SBRT (yes vs no)
9. Invasive mediastinal staging prior to SBRT (yes vs no).
   1. If yes EBUS vs Mediastinoscopy vs other
10. Smoking Status (Current, Ex-smoker, Never smoker).
    1. If a smoker, Pack-Year History

**SBRT data**

1. Prescription dose (e.g 34 Gy in 1 fraction)
2. # of fractions
3. Maximum dose
4. Treatment Start date

**Surgical Minimum Dataset:**

1. Resection type & date
   1. Wedge, segment, lobe
   2. Minimally-invasive, open
   3. LN dissection (at least 3 N2 & 1 N1 stations, recommended)
2. Complications : Ottawa Thoracic Morbidity & Mortality Classification system ([https://ottawatmm.org/#classify](https://webmail.manitoba-ehealth.ca/owa/redir.aspx?C=lhoo7nHSw3q8iTMU0U_Dy8LyWzQHceFwgg25SL7fuO-E1YQee9nXCA..&URL=https%3A%2F%2Fottawatmm.org%2F%23classify)).
   1. This is a validated system modeled after the Clavien-Dindo system and specific to Thoracic Surgery. All major Canadian Thoracic Surgery programs currently use this classification system to evaluate surgical complications.

**Outcomes**

Adverse Events or complications:

Severity of adverse events will be evaluated using the Common Terminology Criteria for Adverse Events (CTCAE) v5.0 grading scale ([https://ctep.cancer.gov/protocoldevelopment/electronic_applications/docs/CTCAE_v5_Quick_Reference_5x7.pdf](https://webmail.manitoba-ehealth.ca/owa/redir.aspx?C=4U-_bV4X8zDs1SXc345wOPhAOo-xnm-OYg5tgTXXws-E1YQee9nXCA..&URL=https%3A%2F%2Fctep.cancer.gov%2Fprotocoldevelopment%2Felectronic_applications%2Fdocs%2FCTCAE_v5_Quick_Reference_5x7.pdf))
